# Directional Control Mechanisms in Multidirectional Step Initiating Tasks

**DOI:** 10.3389/fnhum.2020.00178

**Published:** 2020-07-21

**Authors:** Yuki Inaba, Takahito Suzuki, Shinsuke Yoshioka, Senshi Fukashiro

**Affiliations:** ^1^Department of Sport Science, Japan Institute of Sports Sciences, Japan High Performance Sport Center, Tokyo, Japan; ^2^Graduate School of Human Sciences, Kanagawa University, Kanagawa, Japan; ^3^Japan Society for the Promotion of Science (JSPS), Tokyo, Japan; ^4^Department of Life Sciences, The University of Tokyo, Tokyo, Japan

**Keywords:** anticipatory postural adjustment (APA), multidirectional steps, center of pressure (COP), center of mass (COM), gait initiation, electromyography (EMG)

## Abstract

Typical anticipatory postural adjustments (APAs) in forward gait or step initiation tasks to prepare for possible disturbances caused by prime voluntary movements and to accelerate the body forward have been previously reported. However, it is not clear how wide the variations in step directions are differentiated and controlled in non-forward step initiation tasks during the APA phase. The main goal of this study is to explain the directional control mechanisms by investigating the APA of step initiation tasks in forward, diagonal, lateral, and posterior directions. The center of pressure (COP) trajectories and related muscle (soleus, tibialis anterior, and gluteus medius of both lower limbs) activities during the APA of step initiation tasks in nine different directions were analyzed in six healthy young males. Posterior shifts of COP during APA decreased as the direction became more lateral (0° to 90°). For posterior step initiations, COP moved anteriorly from the initial position to accelerate the center of mass of the whole body (COM) backward. Lateral shifts of COP toward the stepping foot during APA decreased as the stepping direction became more lateral (from 0° to 45° and from 180° to 113°) while it plateaued to about zero in the direction from 45° to 113°. Both anteroposterior and lateral displacements of COP in APA were nonlinearly modulated to each direction, but they were linearly related to the anteroposterior and mediolateral component of the velocities of COM at the take-off of the stance foot. Thus, the scaling of APA, reflected in the anteroposterior and lateral displacements of COP and the temporal sequence of selected muscle activities, was based on the anteroposterior and mediolateral components of the take-off velocity of COM that ultimately controls the direction of steps.

## Introduction

Step or gait initiation from a quiet stance is a challenging task that requires a balance control to move from the static to the dynamic state (Yiou et al., 2017). To accomplish this biomechanically challenging task, typical muscle activities (Carlsöö, 1966; Mann et al., 1979; Crenna and Frigo, 1991; Elble et al., 1994; Lepers and Brenière, 1995) and the movement of the center of pressure (COP) under feet (Yamashita and Katoh, 1976; Brenière and Do, 1986; Jian et al., 1993) occur before the lift-off of the stepping foot (foot leaving the floor first). These involuntary activities observed before prime or intended movements are conventionally termed “anticipatory postural adjustments (APAs)” and regarded as the reflection of motor programs for executing intended voluntary movements (Crenna and Frigo, 1991).

APAs originally refer to postural adjustments that are performed before prime voluntary movements to minimize the disturbances caused by the subsequent movements (Belen’kiĭ et al., 1967; Bouisset and Zattara, 1987; Massion, 1992). For instance, when lifting an upper arm rapidly, modulations in muscle activities of the lower limbs, pelvis, and trunk occur before the activation of the anterior deltoid, which is the prime mover of the upper arm, to maintain the whole-body balance when the prime motion is performed (Zattara and Bouisset, 1988). For initiating the gait, if the subject lifts off the stepping foot without any prior postural adjustments, the body becomes unstable because the center of mass of the whole body (COM) is off the base of support. Therefore, before lifting the stepping foot, COP is shifted toward the stepping foot first and COM is accelerated toward the stance foot (the last foot to push off and leave the floor) to avoid a side-fall and successfully initiate the gait forward (Jian et al., 1993). Then, COP is shifted toward the side of the stance foot to transition to a single stance to push off the floor. The APA for gait initiation is also characterized by the deactivation of the soleus (SOL) and gastrocnemius muscles followed by the activation of the tibialis anterior (TA) muscles of both lower limbs, which result in the backward shifts of COP (Cook and Cozzens, 1976). This backward shift of COP generates a disequilibrium between COP and COM and accelerates the COM forward. Thus, these typical APAs are necessary for not only avoiding falls but also for propelling COM in the forward direction at forward gait initiation.

In addition to the forward gait initiation, attention should be directed to step initiation mechanisms in non-forward directions. In fact, 10–50% of the steps performed daily are in non-forward directions (Glaister et al., 2007). About 25% of the steps taken during defensive and offensive moves in soccer are in non-forward directions such as backward, diagonal, and lateral directions (Bloomfield et al., 2007). Though some studies investigated perturbation-induced steps to forward, backward, and lateral directions (e.g., Tripp et al., 2004; Gray et al., 2017), only a few studies have investigated the APAs of voluntary non-forward steps. Lyon and Day (1997) studied the mediolateral postural control before the toe-off of the stepping foot in forward and diagonal steps both experimentally and using simulations. The experimental results showed that the extent of the lateral positional shifts and the velocity of COM toward the stance foot at the time of the toe-off of the stepping foot varied with the stepping direction. The trajectory of COM after the toe-off of the stepping foot was closely predicted by a single-segment mathematical model that freely falls only under the influence of gravity when initial COM positions and velocities were set based on the experimental values adjusted by the directions. The results indicated the need for mediolateral postural control by the time of the toe-off of the stepping foot according to the stepping direction. These adjustments of the extent of mediolateral COM shift and velocity toward the stance foot were assumed to be achieved by modulating the displacement of COP toward the stepping foot as their relationship was reported in forward gait initiation (Jian et al., 1993). A previous study investigated the multidirectional gait initiation in the directions of −15°, 0°, 15°, and 30° (0° is forward, negative values are toward the direction of the stance foot, and positive values are toward the direction of the stepping foot) under slow (normal) and fast speed conditions and reported that the amplitude of lateral shifts of COP toward the stepping foot during the APA phase was correlated to the desired stepping direction (Corbeil and Anaka, 2011). The results of the previous studies suggest that adjusting the extent of the mediolateral shifts of COP and consequently the mediolateral COM position and velocities by the time of the toe-off of the stepping limb or the end of the APA phase is required for differentiating the stepping directions in forward and diagonal steps.

In contrast to the well-established role of the mediolateral COP shifts during APA in directional control, the role of the anteroposterior shifts of COP during APA is still debated. Intrinsically, the horizontal direction, by its physical definition, is determined by anteroposterior velocity and mediolateral velocity. Previous studies on forward gait initiation showed that the amplitude of posterior shifts in COP during APA was positively correlated with the anterior velocity of the COM at the end of the first step (Brenière et al., 1987; Lepers and Brenière, 1995). Another study reported that the amplitude of the TA activity during APA, which was correlated to the posterior shifts of COP, was greater in the forward gait initiation with a greater movement velocity (Crenna and Frigo, 1991). Based on these results, anteroposterior shifts of COP may also be related to the anteroposterior velocity of COM in multidirectional step initiation tasks and consequently is related to the direction of steps. An experimental study on diagonal steps whose directions were not more than 30° from the anterior direction reported that the stepping direction showed a weak association with the amplitude of COP posterior shifts (Corbeil and Anaka, 2011). These results raise the question of why the relationship between the stepping direction and anteroposterior shifts of COP has not been detected in multidirectional step initiation tasks although the anterior velocity of COM at the end of the steps is related to the posterior shifts of COP in forwarding steps.

One of the reasons for the detection of only the weak associations between posterior shifts of COP during APA and the direction (Corbeil and Anaka, 2011) is related to the tested range of stepping directions. For instance, when the anteroposterior axis, whose anterior direction is set to 0° and positive, and the mediolateral axis, whose positive direction is 90° counterclockwise, are defined, mediolateral and anteroposterior components of the velocity of COM are given as sine and cosine of the directions of the steps multiplied by the speed on the horizontal plane, respectively. The difference between the sine values of −15° and 30° is ~0.76 and that between the cosine values of 0° and 30° is ~0.13. Thus, when tested at this range, where a greater variation in the mediolateral components than in the anteroposterior components of the velocity of COM at the end of the steps occurs, the relationship between the mediolateral shifts of COP and the step direction or the mediolateral velocity of COM was detectable while the relationship with anteroposterior components was not. Therefore, if steps to the directions greater than 30°, of which the cosines have greater differences, were included, greater changes in anteroposterior components of the velocity would occur. Then, the relation between the anteroposterior shift of COP during APA and anteroposterior velocity of COM at the toe-off of the stance foot and the stepping direction could be detectable. Therefore, we hypothesize that not only lateral but also anteroposterior shifts of COP during APA in multidirectional steps are related to control the direction of the steps. To test this hypothesis, we investigated the COP, the velocity of COM, and the muscle activity of lower limbs during the steps at the maximum effort in nine directions ranging from forward to backward movements on the side of the stepping foot.

## Materials and Methods

### Experimental Design

Six healthy male subjects (age: 28 ± 4 years; height: 170.1 ± 4.8 cm; body mass: 70.0 ± 4.1 kg; mean ± standard deviation) participated in the experiment. The present study was approved by the ethics committee of the University of Tokyo. The subjects read the description of the purpose, risks, and basic procedures of the experiment and gave their written informed consent for participation.

Maximum voluntary contraction (MVC) tasks utilizing manual loads (Hislop and Montgomery, 2002) were performed before stepping trials to record the maximal electromyographic (EMG) amplitude for each muscle being tested. MVC tasks for each targeted muscle were performed twice for at least 3 s. Then, the stepping trials were performed. Stepping and stance feet for all subjects were specified to be the left and the right foot, respectively. At first, the subjects performed the forward step as they perform it in their daily life. The step distance from the center of the feet at the initial standing position to the center of the feet after the forward step initiation was measured. The measured step distance was marked with pieces of color tape on the floor in all directions. Then, subjects performed step initiation tasks in nine directions as fast as possible to reach the marked distance after familiarization. Nine directions were 0° (forward), and 23, 45, 67, 90, 113, 135, 157, and 180° left (toward the side of the stepping foot) from 0°. The subjects undertook the steps without arm swings (hands were kept on the sides of the lower back) and faced forward throughout the trial. The order of the steps was randomized and five steps were performed in each direction. The direction of the trial was communicated to the subjects before the trials. They stood still until a light-emitting diode (LED) placed in front of them lit up to initiate a step.

### Data Collection

Two force plates (9281B, Kistler, Switzerland), with each foot on a force plate, were used to measure the ground reaction forces (GRF) at 1,000 Hz. A footswitch system (PTS-112A, DKH, Japan) with a pressure-sensitive sensor (PH-463, DKH, Japan) was attached to the heel of the stepping foot to detect the instant when the heel left the floor. The EMG activities of the TA, SOL, and gluteus medius (GMED) muscles of the stance limb and the stepping limb were monitored using the wireless system (WEB-1000, Nihon Kohden, Japan) at 1,000 Hz.

### Data Analysis

The APA phase was defined as the phase from the moment when the LED lit up to the moment of take-off of the heel of the stepping foot, which was detected from the obtained footswitch data. The net COP or the position of COP of both feet was calculated using the COP recorded by the force plates under each foot (Jian et al., 1993). In this article, COP refers to the net COP unless stated otherwise. The maximal anteroposterior and lateral displacements of COP during APA from the initial position were computed. Positive values in maximal anteroposterior displacements mean that the COP moved forward from the initial position and negative values mean it moved in the backward direction. For lateral displacements, only the displacements toward the stepping foot (to the left in this study) from the original position were measured and reflected as positive values; the displacements toward the stance foot were not computed as negative values because COP eventually moved to the stance foot in all trials. The velocities of COM at the heel-off of the stepping foot and toe-off of the stance foot were calculated to represent the velocities at the end of the APA phase and the step, by integrating the GRF of each component from the moment of the LED lit-up to the moment of the heel-off of the stepping foot and toe-off of the stance foot which was determined by the footswitch and vertical component of GRF data, respectively. Linear correlations were tested between the anteroposterior and mediolateral components of COM velocity at the heel-off of stepping foot and the toe-off of the stance foot, and maximal anteroposterior and lateral shifts of COP during the APA phase.

Raw EMG data signals (MVC and stepping trials) were high-pass filtered at the cut-off frequency of 20 Hz (Jacobs and van Ingen Schenau, 1992). The EMG signals were then full-wave rectified. The rectified EMG signals of MVC tasks and stepping trials were averaged (average rectified values: ARV) into 1 s intervals and the APA phase, respectively. The highest value of the 1-s ARV among two MVC tasks was used to normalize the ARV of the APA phase of stepping trials.

In the analysis, the mean of the data (maximal anteroposterior and lateral COP displacements, anteroposterior and mediolateral components of COM velocity at the end of APA phase and toe-off of stance foot, and ARVs) of the five steps was used as the representative value of the step condition of each subject.

### Statistical Analysis

A one-way repeated-measures analysis of variance was conducted to test the effect of direction on maximal anteroposterior and lateral COP displacements and ARV. The Greenhouse-Geisser degrees of freedom correction was used to correct for the violation of the sphericity assumption (Winer et al., 1991). When the effect of direction was found, *post hoc* multiple comparison Shaffer’s tests (i.e., Shaffer, 1986) with Holland-Copenhaver constants (Holland and Copenhaver, 1987) were conducted for establishing differences between step directions. The Pearson correlation coefficient was used to establish the relationships between maximal anteroposterior and lateral displacements of COP and the anteroposterior and mediolateral velocities of COM at the toe-off of the stance foot and at the end of the APA phase or the heel-off of the stepping foot. The level of statistical significance was set at *P* < 0.05.

## Results

**Figure 1:**
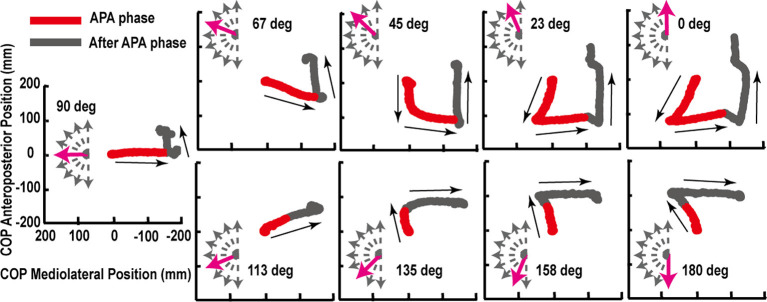
Typical example of the center of pressure under feet (COP) trajectories for multidirectional step initiation tasks. The red lines show the COP trajectories during anticipatory postural adjustment (APA; red) phase or the phase from the lit-up of starting light-emitting diode (LED) to the take-off of the heel of the stepping foot. The gray lines show the trajectories from the end of the APA phase to the toe-off of the stance foot (after APA phase). The start of the COP anteroposterior and mediolateral position was set to zero.

**Figure 2:**
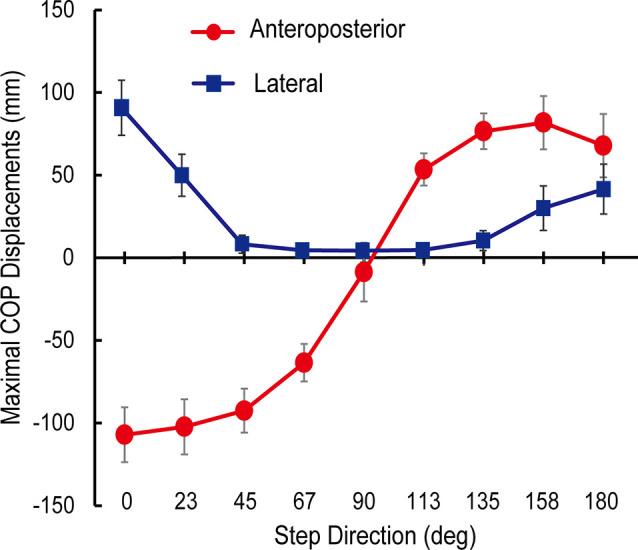
Maximal anteroposterior (red) and lateral (blue) displacements of the center of pressure under feet (COP) during anticipatory postural adjustment (APA) phase from COP position at a quiet stance (mean ± standard deviation of all subjects). Both maximal anteroposterior and lateral COP displacements were significantly different between the stepping directions (*P* < 0.05).

**Figure 3:**
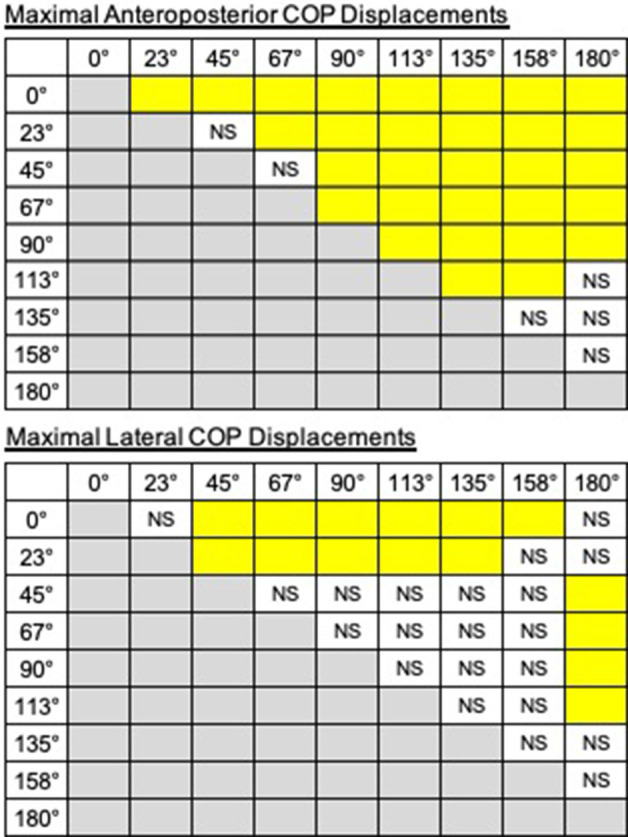
The results of *post hoc* tests on displacements of COP (top: anteroposterior, bottom: lateral). The pairs are shown in the columns colored in yellow show significant differences (*P* < 0.05). NS, non-significant.

The trajectories of COP and the activities of TA, SOL, and GMED on both sides of the lower limbs during the APA phase were adjusted according to the step initiating directions. By initiating the steps in the direction of 0°, COP moved posterior and toward the stepping foot during the APA phase (Figure 1). As the step initiating directions became more lateral, posterior, and lateral COP displacements toward the stepping foot decreased (Figure 1). At the 90° condition, almost no posterior shifts of COP occurred during the APA phase. When the stepping direction was greater than 90° (backward), a forward shift of COP during the APA phase was observed. COP lateral displacements toward the stepping foot, which disappeared at 45° to 113° directions, reoccurred at 135°, 158°, and 180° directions. The trajectories of COP were quantitatively evaluated as the maximal anteroposterior and lateral displacements of COP for all subjects (Figure 2). Statistical tests revealed that the tendency observed in a typical example shown in Figure 1 was consistent for all subjects. A significant effect of the step initiating direction was detected in both maximal anteroposterior and lateral displacements of COP (anteroposterior: *F*_(2.47,12.36)_ = 176.73, *P* < 0.001, partial *η*^2^ = 0.97, *post hoc*: Figure 3; lateral: *F*_(2.34,11.69)_ = 60.28, *P* < 0.001, partial *η*^2^ = 0.92, *post hoc*: Figure 3).

**Figure 4:**
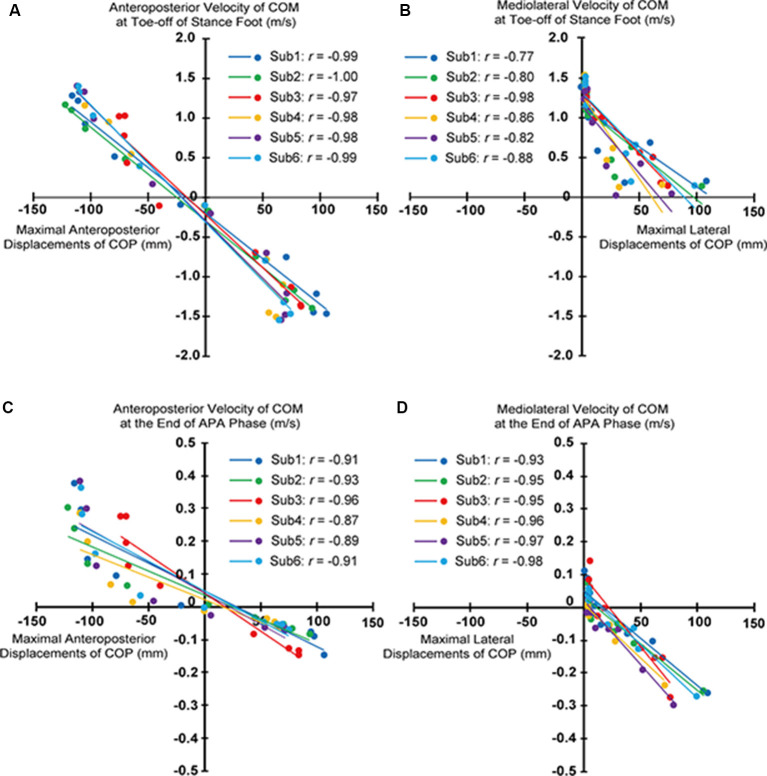
Relationship of the maximal anteroposterior and lateral center of pressure under feet (COP) displacements and anteroposterior and mediolateral components of velocity of the center of mass of whole-body (COM) at toe-off of stance foot **(A,B)** and at heel-off of stepping foot **(C,D)** for each subject.

Significant correlations between maximal anteroposterior displacements of COP and anteroposterior velocities of COM at toe-off of stance foot and those at heel-off of stepping foot, and between maximal lateral displacements of COP and mediolateral velocities of COM at toe-off of stance foot and those at heel-off of stepping foot existed for all subjects (Figures 4A–D).

**Figure 5:**
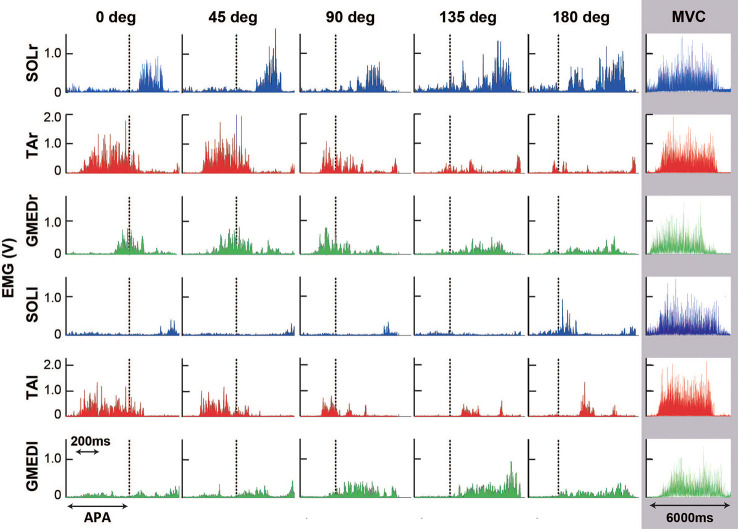
Typical example of rectified electromyographic (EMG) activities of the soleus (SOL; blue), tibialis anterior (TA; red), and gluteus medius (GMED; green) of stance foot side (r) and stepping foot side (l) during steps to 0, 45, 90, 135, and 180 degrees and maximal voluntary contraction (MVC) trial. Dash lines for each figure show the end of the anticipatory postural adjustment (APA) phase. Note that the scale of the horizontal axis (time) is different between the stepping trials (five figures from the left) and the MVC trial (rightmost row) since the difference in movement duration was large.

**Figure 6:**
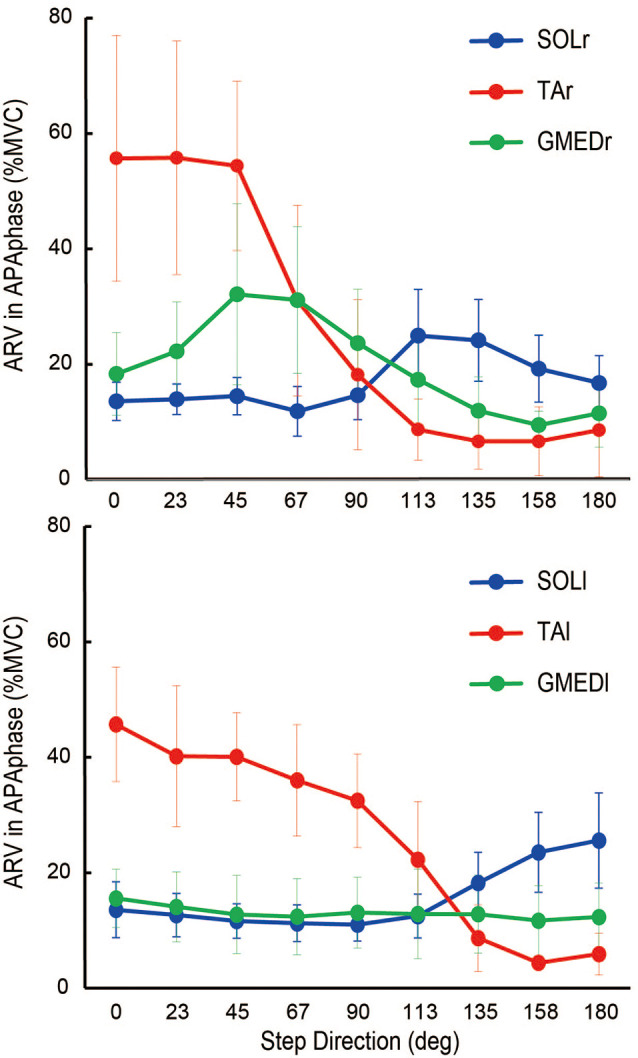
Average rectified value (ARV) of electromyographic activity during APA phase of the soleus (SOL; blue), tibialis anterior (TA; red), and gluteus medius (GMED; green) of stance foot side (r) and stepping foot side (l) during multidirectional steps (mean ± standard deviation of all subjects). The values were normalized by ARV during maximal voluntary contraction (MVC) for each muscle. SOLr, TAr, and GMEDr are shown in the top figure, and SOLl, TAl, and GMEDl are shown in the bottom figure. The muscle activities were modulated according to the direction of steps (*P* < 0.05).

**Figure 7:**
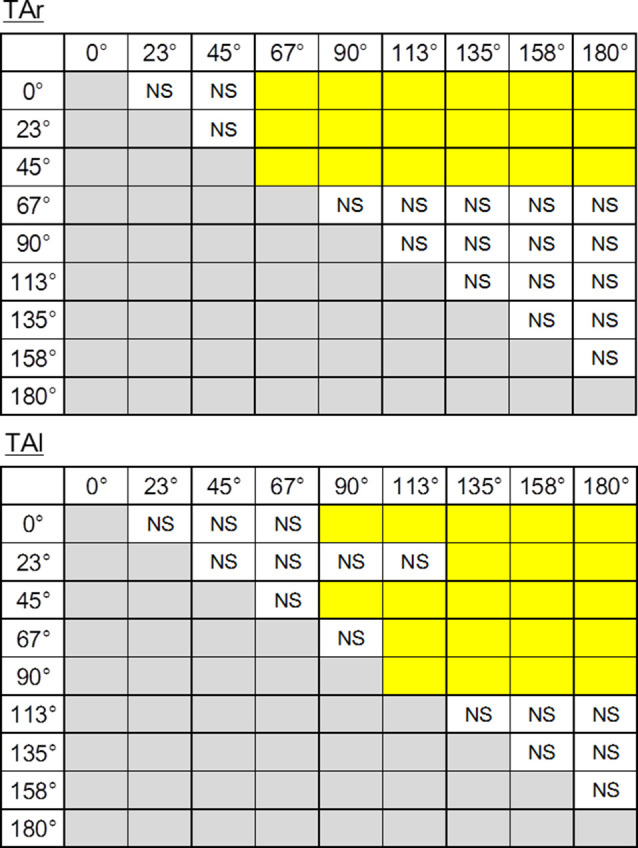
The results of *post hoc* tests on ARV of electromyographic activity during the APA phase of TAr (top) and TAl (bottom). The pairs shown in the columns colored in yellow show significant differences (*P* < 0.05). NS, non-significant.

Temporal patterns of selected muscle activities were modulated according to the step initiating directions (Figure 5). For step initiations in the direction of 0°, the activation of the TA of the stance limb (TAr) and the TA of the stepping limb (TAl) were observed while the activities of SOL of the stance limb (SOLr) and the stepping limb (SOLl) were kept low during the APA phase. As the direction of steps became more lateral and posterior, the activities of TAr and TAl reduced, and the activities of SOLr tended to be slightly increased for step initiations in the directions greater than 90°. The activities of SOLl in the APA phase for posterior directions tended to be greater than those for forward and lateral directions. The activation of GMED of the stance limb (GMEDr) occurred near the end of the APA phase in all directions. As for the GMED of the stepping limb (GMEDl) during APA, the influence of the stepping direction was not consistent across all subjects. Some subjects (two out of six) showed a tendency to decrease the GMEDl activity for lateral steps compared to forward steps while others kept or slightly increased the activities. A significant effect of direction was observed in the ARV of TAr (*F*_(1.93,9.65)_ = 41.72, *P* < 0.001, partial *η*^2^ = 0.89, *post hoc*: Figure 7), SOLr (*F*_(1.46,7.32)_ = 9.86, *P* = 0.011, partial *η*^2^ = 0.66), GMEDr (*F*_(2.24,11.21)_ = 8.48, *P* = 0.005, partial *η*^2^ = 0.63), TAl (*F*_(2.64,13.22)_ = 47.40, *P* < 0.001, partial *η*^2^ = 0.91, *post hoc*: Figure 7), and SOLl (*F*_(1.20,5.98)_ = 11.73, *P* = 0.012, partial *η*^2^ = 0.70; Figures 6, 7) but not in GMEDl (*F*_(2.31,11.57)_ = 2.41, *P* = 0.128). The *post hoc* tests did not find significant differences in the pairs of directions on the activities of SOLr, GMEDr, SOLl.

**Figure 8:**
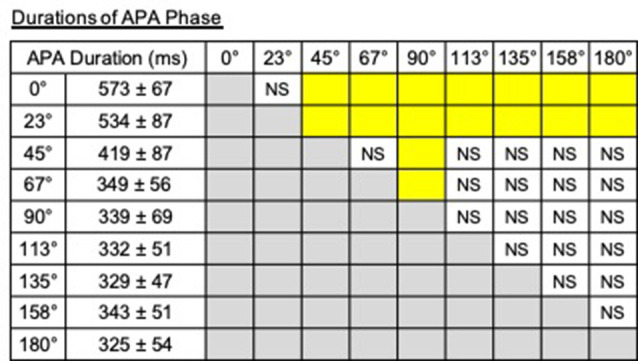
Durations of the APA phase and the results of *post hoc* tests. The pairs shown in the columns colored in yellow show significant differences (*P* < 0.05). NS, non-significant.

A significant effect of direction on the duration of APA phase was observed (*F*_(2.42,12.08)_ = 76.57, *P* < 0.001, partial *η*^2^ = 0.94, *post hoc*: Figure 8). Especially, the APA durations for the steps to 0° and 23° were longer than those to other directions, and that for the steps to 90° was significantly shorter than those for the steps to 0°–67°.

## Discussion

Both maximal anteroposterior and lateral displacements of COP during the APA phase were modulated according to the amplitude of the anteroposterior and mediolateral components of COM velocities at the toe-off of the stance foot, respectively, which ultimately resulted in controlling the direction of the steps. The activities of TA, SOL, and GMED, which were related to the anteroposterior and lateral shifts of COP, were also modulated according to the stepping directions during APA. These results indicate that COP trajectories during the APA phase are scaled as a function of COM velocities at the toe-off of the stance foot or the stepping directions, modulated by the related muscle activities during APA. Although the number of subjects in this study was relatively low, the reported effect size and the *p*-value support the conclusion of this study.

### Adjustment of Anteroposterior Components of COM Velocity Through the Modulation of Corresponding Components of COP Trajectory

Significant correlations between the maximal anteroposterior COP displacements during APA and the anteroposterior components of velocities of COM at the toe-off of the stance foot were observed in multidirectional step initiation tasks (Figure 4A). A previous study reported that the posterior shifts of COP during the APA phase generate uncoupling between the position of COP and COM, leading to the forward acceleration of COM needed for initiating the gait in the forward direction (Jian et al., 1993). Also, the maximal posterior displacement of COP during the APA phase was reported to be correlated to the anteroposterior velocity of COM at the end of the gait initiation toward the forward direction (Crenna and Frigo, 1991; Lepers and Brenière, 1995). Thus, the magnitude of uncoupling between COP and COM in the anteroposterior direction during APA, modulated by the extent of anteroposterior shifts of COP, should influence the anteroposterior acceleration of the COM and eventually the velocity of the COM in multidirectional steps as well.

The modulated anteroposterior velocities of COM at the end of the APA phase being correlated with the anteroposterior shifts of COP during APA were also observed (Figure 4C). However, at both ends of the observed maximal anteroposterior COP displacements, the difference in the velocities of COM at the end of APA phase with respect to the change in COP displacements were observed to be greater than the middle region. It was expected that the different sources used in controlling the COM, muscular or gravitational moment (Lepers and Brenière, 1995) could have contributed to this trend. The greater contribution of muscular moment than the gravity during the APA phase (Lepers and Brenière, 1995) could alter the relationship between COP displacements and COM velocities. If a human body is modeled as an inverted pendulum, or the acceleration of COM is only influenced by the gravity, the distance between COP and COM determines the acceleration given to the COM. On the other hand, if muscular moment does still participate in controlling the movement of COM, then the COP displacements alone cannot predict the velocities of COM. However, considering that the subjects successfully performed steps to the targeted distance and direction, and the linear relationship shown in Figure 4A, it can be said that the position and the velocity of COM at the end of the APA phase met the requirement that they are supposed to have at the end of APA phase, and the human body acted more like an inverted pendulum after APA phase. That is, the modulation during APA phase ultimately affects the anteroposterior components of the COM velocity at the end of the steps. Though this study phenomenologically revealed the correlations between the maximal anteroposterior displacements of COP during APA and the anteroposterior velocities of COM at the end of the steps and the APA phase, further analysis or simulations should be performed to clarify the dynamical model that expresses the relationships between those, such as simulation using an inverted pendulum model. This study, at least, phenomenologically revealed that similar to the velocity control in forward steps, anteroposterior components of COM velocities at the toe-off of the stance foot for multidirectional steps were also modulated through the magnitude of the anteroposterior shifts of COP during APA.

Modulated anteroposterior displacements of COP during APA, which influenced the anteroposterior velocity of COM at the toe-off of the stance foot, ultimately led to the directional control because the direction of steps is determined by the proportion of anteroposterior and mediolateral components of COM velocity at the toe-off of the stance foot. Corbeil and Anaka (2011) reported that the anteroposterior shift of COP was not related to the stepping directions by investigating the step initiations in directions −15° to 30°. The difference in amplitudes of the anteroposterior shifts of COP for the same stepping directions in this study was also very small because the anteroposterior velocity was determined as a function of speed times the cosine of the angle of the stepping direction; the cosine of the angle of the directions of −15° to 30° does not vary significantly in this region. The results of this study improved the understanding of the role of the anteroposterior COP shifts during APA in directional control by investigating the step initiations in wider directions including the region where greater changes in cosine occur.

### Adjustment of Mediolateral Components of COM Velocity Through the Modulation of Corresponding Components of COP Trajectory

Significant correlations between the maximal displacements of COP during APA and the mediolateral component of the COM velocity at the toe-off of the stance foot and the heel-off of the stepping foot were observed (Figures 4B,D). These relations imply that the lateral shifts of COP were also modulated depending on the mediolateral component of the COM velocity. The observed associations suggest that the lateral displacement of COP during APA toward the stepping foot would not “generate” the lateral velocity but “suppress” the potentially generated lateral velocity, as it was reported in forward and diagonal steps by the previous study (Lyon and Day, 1997). COM would be accelerated toward the side of the stepping foot if COP was not shifted toward the stepping foot first during APA in forward gait initiation; the archetypal role of the COP shifts toward the stepping foot was to accelerate the COM toward the stance foot and prevent the body from toppling down toward the side of the stepping foot to successfully step forward (Lyon and Day, 1997). As the stepping direction became more lateral (from 0° to 45° and from 180° to 113°), the magnitude of the maximal lateral COP displacement decreased (Figure 2). This finding is consistent with the assumption that the magnitude of the maximal lateral COP displacement was scaled to the amplitude of the mediolateral velocity that needed to be suppressed. These suppressing activities were reflected in the COM velocities at the time of heel-off of stepping foot; negative COM velocities or toward the stance foot side were generated when COP was shifted toward the stepping foot side (Figure 4D). When stepping forward, since the mediolateral velocity of COM at the toe-off of the stance foot should be zero, COP is shifted toward the stepping foot to generate the velocity of COM toward the stance foot side. This cancels the mediolateral velocity toward the stepping foot that could have been potentially developed by lifting the stepping foot without the lateral COP shift. For stepping diagonally in the 23° direction, since mediolateral velocity in the stepping direction must be generated, naturally developed mediolateral velocity toward the stepping foot side was only partially suppressed by developing the velocity toward the stance foot, modulated by the COP shifts toward the stepping foot. For steps to the 45–113°direction, the maximal lateral COP displacements almost plateaued; no suppression was required but the mediolateral velocity that would be naturally generated was utilized to generate the required mediolateral velocity of COM at the toe-off of the stance foot (possibly the additional mediolateral velocity has to be generated in addition to the mediolateral velocity generated by APA activities to satisfy the required mediolateral velocity for stepping to the intended direction). For the backward steps to 135°–180°, lateral shifts of COP toward the stepping foot to accelerate COM toward stance foot side, which was reported by a previous study that investigated compensatory backward steps to perturbation (McIlroy and Maki, 1999), were also observed. The greater amplitude of lateral shifts of COP in the steps to 0° and 23° was also reflected in the greater APA durations, which suggests that those were more challenging in terms of the reported functional role of controlling mediolateral postural stability (Yiou et al., 2012, 2017) compared to other directions (Figure 8).

The relationship between the maximal lateral COP shifts during APA and the stepping direction was not linear in our study (Figure 2), whereas its linearity had been reported by a previous study (Corbeil and Anaka, 2011). This inconsistency was attributed to the investigated region of stepping directions. The previous study was limited to the narrower variation of directions, which correspond to the direction of 0–30° in our study. In this region, the relationship can be deemed as linear (Figure 2). By expanding the range of directions, this study showed the nonlinear relationship between the maximal lateral shifts of COP and the direction of the steps, linear relationship between the amplitude of lateral shifts of COP and the mediolateral components of velocities of COM at the heel-off of stepping foot, and although indirect, the linear relationship between the amplitude of lateral shifts and the mediolateral components of velocities of COM at the toe-off of the stance foot.

### Modulation of the Muscle Activities During APA Phase

The modulation in the amplitude of the activities of muscles related to APA in gait initiation coincided with the nonlinear pattern in anteroposterior and lateral COP displacements modulated according to the directions of the steps. The most remarkable modulations were observed at the ankle dorsiflexors and plantar flexors. COP posterior shifts are controlled by the combination of the activation of TA and the deactivation of plantar flexor muscles, such as the SOL (Crenna and Frigo, 1991; Elble et al., 1994). The TA activities of both sides during APA increased with the forward movement velocity in forward step initiation tasks (Crenna and Frigo, 1991). For multidirectional step initiation tasks, the required anterior velocity of COM at the toe-off is greater in forward steps than in lateral steps and becomes smaller in posterior steps (turns to negative in posteriorly directed velocity). Accordingly, a decrease in the activities of TAr and TAl was observed during APA phases as the direction of the step initiations became more lateral and posterior in this study (Figures 5, 6). A slight increase in the activities of SOLr (stance foot side) with the increased step direction to greater than 90° was also observed (Figures 5, 6). For stepping laterally and posteriorly, subjects kept activating or slightly increased the activities of SOLr during APA in contrast to that in forward steps, where the activation level of SOLr was kept relatively low, which was also observed in forward steps in a previous study (Cook and Cozzens, 1976). The combination of continuous SOLr activity and decreased TA activity contributed to the increase in the ankle plantarflexion moment and the decrease in the amplitude of COP posterior shifts, reducing the anterior component of COM velocity at the toe-off in laterally or posteriorly directed steps.

Muscle activity modulations related to lateral shifts of COP were more complicated and inconsistent between the subjects than those related to anteroposterior shifts. Originally, the activation of the stepping limb SOL (Elble et al., 1994) and stepping limb GMED (Carlsöö, 1966; Mann et al., 1979; Winter, 1995; Mickelborough et al., 2004) have been reported to contribute to the shifting of COP toward the stepping limb in forward gait initiation. Honeine et al. (2016) proposed that the activation of the stance limb TA during the APA phase flexes the knee joint and unloads the stance limb, shifting the COP toward the stepping foot in combination with the activation of the stepping limb hip abductors. If TAr (stance foot side in this study) plays a role in shifting the COP toward the stepping foot in multidirectional steps, the activities of TAr would be decreased in lateral direction steps, where maximal lateral displacements toward the stepping foot also decrease. A decrease in the activities of TAr during APA was observed with the decrease in the lateral COP displacement for lateral steps. Although SOLl (stepping foot side in the present study) was expected to decrease for the lateral steps to suppress the COP lateral shifts since the activity of SOLl was positively related to the lateral shifts of COP toward the stepping foot (Elble et al., 1994), that tendency was not observed in this study. These expected results for TAr and unexpected results for SOLl are attributed to the possible dual role of TAr and SOLl in influencing both the anteroposterior and mediolateral COP trajectories. As for TAr in more lateral steps, a decrease in activity is desired for satisfying the demand to decrease lateral shifts of COP toward the stepping foot and to decrease posterior shifts. Thus, the demands for TAr to modulate the anteroposterior and mediolateral displacements of COP coincided. In contrast, SOLl was required to decrease the activity to reduce the lateral shifts of COP, whereas it was required to increase the activity to reduce posterior shifts; the demands for SOLl were the opposite of those for TAs in modulating lateral and anteroposterior COPs. In posterior steps, however, since the demand for SOLl to shift COP laterally toward the stepping foot and forward coincided, the slight increase in activity at the end of the APA was observed. Therefore, in multidirectional steps, the extent of modulation of the activities of plantar flexors and dorsiflexors are influenced by independent components of anteroposterior and lateral displacements of COP and by both components at the same time.

As for the ARV of GMEDl, a reduction in lateral steps and return in posterior steps, which were expected to be observed considering the tendency in COP mediolateral displacements, was not observed (Figure 6). Honeine et al. (2016) reported that some of the subjects in their study relied more on the stepping limb hip abductor and others on the stance limb dorsiflexor activity [which Honeine et al. (2016) associated to knee flexion] while most relied equally on both to control the mediolateral shifts of COP. Similarly, in this study, some subjects (two out of six) showed a tendency to decrease the GMEDl activity for lateral steps compared to forward steps. Thus, the variations in choosing the muscle activity for mediolateral shifts of COP resulted in an insignificant difference observed for GMEDl in this study. As for GMEDr, the activation at the end of the APA phase contributed to the return of the COM toward the stepping foot direction, as well as to the preparation to stabilize the pelvis when the swing foot leaves the floor as reported in the previous study that investigated forward gait initiation (Mickelborough et al., 2004). These combinations of modulation of selected muscles in the APA phase contributed to the control of the COP shifts and COM velocity and the ultimate control of the stepping directions. However, to further discuss the relationship between muscle activity and mediolateral COP trajectories, future research should include the investigation of other muscle groups and more variations in lateral directions.

### Possible Influence of Resultant COM Velocity on the Motor Program Reflected in APA During Multidirectional Steps

As the result of this study revealed, since the COP trajectory during the APA phase is controlled depending on the amplitude of required resultant COM velocity at the toe-off of the stance foot, the APA pattern can be different even in the steps in the same direction. In the steps to the directions 45°–113°, COP moved directly toward the stance foot in this study (Figure 1). On the contrary, Tateuchi et al. (2011) reported small lateral COP shifts toward the stepping foot during the APA phase for small (the distance was 20 cm at maximal) lateral (90°) steps in elderly people and elderly patients with hip osteoarthritis. The difference in the stepping distance (in this study, it was about 70 cm) is assumed to have influenced the COP trajectory pattern even in the steps toward the same lateral directions since it requires different amplitude of resultant COM velocity at the toe-off of the stance foot. As Lyon and Day (1997) reported, the lateral shifts of COP toward the stepping foot suppress the COM velocity toward the stepping foot that would be generated if no COP adjustments were performed. Therefore, for stepping laterally toward the stepping foot side as fast as possible to the naturally stepped distances as performed in this study, the subjects did not shift COP laterally toward the stepping foot since it would slow down the stepping movement. They rather preferred to adopt the strategy of utilizing the naturally generated lateral velocity toward the stepping foot side by lifting the stepping foot without shifting COP toward the stepping foot side to generate velocity toward stepping side at an earlier stage. On the other hand, the elderly subjects who were instructed to step only short distances did not want to generate as much lateral velocity toward the stepping foot side as naturally generated by lifting off the stepping foot without shifting COP toward the stepping foot, but rather needed to adjust the lateral velocity so that they can land at the instructed distance. Though the observed pattern was different between these studies, it can be said that the fundamental principle of the role of the APA was consistent; the amount of COP shift is modulated according to the intended resultant COM velocity at the toe-off of the stance foot. However, future studies are needed to confirm if the consistent relationship exists for the steps with various distances leading to different resultant velocities; in particular, it is possible that for generating greater velocities at the toe-off of the stance foot than this study investigated, greater contribution of muscular moments might be required and the relationship could be altered.

Also, the stepping tasks were all directed to leftward with stepping foot as the left side. It is possible that depending on the stepping side preferences or dominant side of participants, the preferred stepping distance and duration it takes for completing the tasks differ. However, based on the results of our study, it is assumed that the differences in those preferences or characteristics of a certain population are reflected in the resultant COM velocities, and APA will be also modulated accordingly. Therefore, though this study recruited only young healthy males, and did not investigate the influence of age, injuries or disease histories, and stepping side preferences, the phenomenological model established by this study can serve as a reference control strategy if future studies investigate those populations with different stepping characteristics.

## Conclusion

We investigated the directional control mechanisms in step initiations to the forward, diagonal, lateral, posterior diagonal, and posterior directions at the same distance. We analyzed the APA, regarded as the reflection of a motor program of planned movement. Both maximal anteroposterior and lateral displacements of COP and related muscle activities during the APA phase were modulated nonlinearly to each direction. This modulation is attributed to the linear relationship between maximal anteroposterior and lateral displacements of COP and the correspondent velocities of COM at the toe-off of the stance foot. Although the maximal lateral displacements of COP were linearly related to the mediolateral velocity of COM at the toe-off of stance foot, they plateaued in the direction of 45°–113° because the lateral shifts of COP were modulated according to the amount of suppression of mediolateral velocity that would be naturally generated without COP lateral shifts toward the stepping foot. These results suggest that the changes in not only lateral but also anteroposterior maximal COP displacements during the APA phase through the modulation of related muscle activities influenced the velocity of COM generated at the time of the toe-off of the stance foot, which ultimately results in controlling the direction of the steps in multidirectional step initiation tasks.

## Data Availability Statement

The datasets generated for this study are available on request to the corresponding author.

## Ethics Statement

This study was carried out in accordance with the recommendations of the ethics committee of the University of Tokyo with written informed consent from all subjects. All subjects gave written informed consent in accordance with the Declaration of Helsinki. The protocol was reviewed and approved by the ethics committee of the University of Tokyo.

## Author Contributions

YI, SY, and SF designed the study. YI collected and analyzed the data. YI, TS, SY, and SF interpreted the data, drafted the manuscript, and gave final approval.

## Conflict of Interest

The authors declare that the research was conducted in the absence of any commercial or financial relationships that could be construed as a potential conflict of interest.
